# Relationships among instructor autonomy support, and university students’ learning approaches, perceived professional competence, and life satisfaction

**DOI:** 10.1371/journal.pone.0266039

**Published:** 2022-04-14

**Authors:** Elisa Huéscar Hernández, José Eduardo Lozano-Jiménez, Jose Miguel de Roba Noguera, Juan Antonio Moreno-Murcia

**Affiliations:** 1 Department of Health Sciences, Miguel Hernández University, Elche, Spain; 2 Human and Social Sciences Faculty, Universidad de la Costa, Barranquilla, Colombia; 3 Department of Sport Sciences-Sport Research Centre, Miguel Hernández University, Elche, Spain; Hanyang University, REPUBLIC OF KOREA

## Abstract

The purpose of this study was to examine relationships among instructor autonomy support for student learning, and students’ motivational characteristics, learning approaches, perceptions of career competence and life satisfaction. Participated 1048 students from Spanish universities with ages between 18, and 57 years. A Structural equation modeling revealed a relationship between instructor autonomy support for student learning with students’ basic psychological need satisfaction. As a result, students’ basic need satisfaction was related to their intrinsic motivation, and to a deeper learning approach. These educational outcomes contributed to explain students’perceived professional competence, and life satisfaction. These findings highlight the importance of student choice, and decision-making in the learning process as a means to facilitating deeper learning, stronger feelings of professional competence, and enhanced well-being.

## Introduction

Current workplace demands necessitate that university students not only acquire theoretical knowledge but that they also develop the capacity to “learn how to learn” in order to have the capacities necessary to adapt to rapidly changing workplace demands in a self-directed manner [1]. As part of the university model that is promoted through current initiatives (*Espacio Europeo de Educación Superior*), the current focus for higher education is grounded in the development of varied student competencies, and proposes methodological approaches to the teaching/learning process in which graduating students have received adequate preparation based in competencies that will be transferable across varied contexts. In addition, specific content-area competencies are needed that will enable success in any given educational, workplace, and social contexts.

In relation to the goal of optimizing the anticipated fit between the academic environment, and the future employment demands that will be placed upon students, it is recommended that the relationship between universities, and professional workplace associations be strengthened through appropriately designed university curricula [2]. Recent work in this area has proposed that student courses of study incorporate this focus, and reformulate the academic competencies with an eye on future professional necessities [3–5]. In essence, the student role would change to become more process-oriented rather than the current content-oriented focus in learning [6]. This outcome could be achieved through a greater appreciation that professional, and personal development must reflect the dynamic nature of the workplace, and the need for individuals to be able to adapt to change [7].

Self-determination theory (SDT) [8] has been a widely used theoretical framework from which to understand student motivation in relation to cognitive/academic, behavioral, and emotional outcomes [9]. It has been proposed by theorists that the satisfaction of basic psychological needs (BPN) will contribute to a host of positive, and adaptive outcomes [10]. One highly relevant influence in the learning environment involves the teacher/student interactional style during the learning process, which is typically considered to reflect the pattern of interpersonal processes that occur between teachers, and students while students carry out their work [11]. From this perspective, classroom interactional practices are nutriments that contribute to the internal motivational resources of the student, and an autonomy-supportive instructional style should be beneficial in the realization of this goal [11]. Research indicates that an autonomy-supportive instructional style is associated with students acquiring knowledge in a reflexive manner, and that this style also increases student participation, self-confidence, self-esteem, commitment, initiative, and enthusiasm for learning [12–15]. These beneficial outcomes reflect a state of meaningful learning [16], and seem to contribute to the improvement of academic performance [17]. To the contrary, instructional styles that reflect teacher control or hostility are associated with maladaptive student outcomes [18].

Self-determination theory presents a continuum of motivational styles that includes intrinsic motivation, extrinsic motivation, and amotivation. The preferred type is intrinsic motivation which is self-determined in nature, and is characterized by the student’s desire to gain knowledge, and to experience stimulation in the learning process. Extrinsic motivation is less self-determined, and ranges among four expressions that are labeled external regulation, introjected regulation, identified regulation, and integrated regulation. These four expressions of extrinsic motivation vary in the extent of self-regulation. A final point on the motivational continuum is amotivation, which refers to the absence of motivation, whether it be intrinsic or extrinsic. At a broader level, motivation can be differentiated between autonomously regulated forms of motivation, which include intrinsic motivation, and identified, and integrated regulation, and controlled motivation which consists of extrinsic motivation, and externally regulated, and introjected forms of motivation [19]. More autonomously regulated forms of motivation should be expected to result from those learning contexts in which choice, and initiative is encouraged from the student [20] as these behaviors seem to drive a sense of satisfaction as the individual develops competencies [21].

Autonomous forms of motivation, and the corresponding satisfaction of basic psychological needs are anticipated to contribute to psychological well-being in students of higher education and to result in greater self-esteem and more favorable academic self-concept whereas controlled forms of motivation inhibit psychological need satisfaction, and have also been linked to anxiety, and lower levels of self-esteem [22]. Meaningful learning ought to be a fundamental component of the approach to the new model of developing academic competency where the student acquires knowledge as the foundation of cognitive processes but in such a way as to lead to a higher level of thinking, and a deeper understanding of interdisciplinary knowledge that is useful, and relevant [13, 23]. Students can also adopt different approaches to learning in accordance with the nature of the academic learning environment [24]. A constructivist approach to learning is considered to be one in which students actively engage in deeper learning processes [25] whereas a superficial approach [26] is used to describe the strategies employed by those who seek to memorize content without making efforts to engage in a broader application of their learning. These learning approaches have also been related to the student’s type of motivation [27], and to the extent of psychological need satisfaction that the student experiences given that when the basic psychological needs (BPN) are satisfied that students will employ a wider variety of learning strategies, adopt fewer avoidance strategies, and be more likely to attain a higher level of academic performance [28, 29].

Some researchers have assessed the relationships among students’ level of autonomy support, their academic competencies, and their approaches to learning from the framework of self-determination theory [30, 31]. However, research has yet to be conducted relative to the influence of autonomy support upon feelings of perceived professional competence, and life satisfaction outcomes. As a consequence of the preceding logic relative to the importance of autonomy support for students in the university phase for the acquisition of competencies, the purpose of this study was to assess the predictive strength of instructor autonomy support, level of basic psychological need satisfaction, and academic motivation on the processes of deep learning, and corresponding effects on perceived professional competence, and life satisfaction of students. It was hypothesized that autonomy support would be positively associated with satisfaction of the basic psychological needs, and with intrinsic motivation. In addition, autonomy support was anticipated to predict students’ approaches to learning which, in turn, would contribute to the explanation of perceived professional competence, and life satisfaction.

## Materials and methods

### Participants

The sample was comprised of 1048 university students, including 365 men (34.8%), and 683 women (65.1%). The participants ranged in age from 18 to 57 years of age (*M* = 22.17 yrs., *SD* = 4.20 yrs.), and attended various Spanish universities, and were engaged in a program of study related to sport, and exercise science or psychology.

### Measures

#### Autonomy support

The Teacher´s Care Scale developed by Saldern, and Littig [32], and validated for use in the Spanish language, and educational context by Moreno-Murcia, Ruiz, Silveira, and Alías [30] was employed to assess instructor support for student autonomy. On this instrument, students respond to the common stem phrase of, “Our teacher…” to questions that relate to students’ perceptions of their instructor’s interest, and involvement in their learning (e.g., “Is concerned about student problems”). This instrument includes four items, and the response format utilizes a Likert-type scale with response choices that range from “1” (“*Never or almost never*”) to “4” (“*Frequently true for me*”). The Cronbach alpha internal consistency value for this scale was .81 in the present study. Assessment of the instrument’s factor structure through confirmatory factor analysis revealed good fit (fit indices of χ2/g.l. = 5.15; *CFI* = .99; *IFI* = .99; *RMSEA* = .06).

#### Basic psychological needs

The assessment of satisfaction of basic psychological needs was conducted through the Basic Psychological Need Satisfaction in Education Scale (*Escala de Satisfacción de las Necesidades Psicológicas Básicas en Educación*) developed by León, Domínguez, Núñez, Pérez, and Martín-Albo [33]. This instrument is a Spanish language modification of the original French language scale by Gillet, Rosnet, and Vallerand [34]. The instrument consists of fifteen items that assess satisfaction of the three basic psychological needs, and the individual’s perceptions of autonomy (e.g., “I feel free to make my own decisions”); competence (e.g., “I feel that I can do things well”); and relatedness (e.g., “I feel good about the people with whom I interact”). Student responses are provided on a five point Likert-type scale that ranges from “1” (“*totally disagree*”) to “5” (“*totally agree*”). The Cronbach alpha values of internal consistency in the present study for the individual dimensions were .76 for competence; .68 for autonomy; and .80 for relatedness. Fit indices in the present study were: χ2/g.l. = 6.8; *CFI* = .91; *IFI* = .91; and *RMSEA* = .074.

#### Academic motivation

To assess student intrinsic motivation for academic work, the Scale of Motivation in Education [35] was used. This scale has been translated from its original French language form, and validated for use in the Spanish language, and cultural context by Nuñez, Martín-Albo, and Navarro [36]. The common stem phrase across all questions is, “Why do you study?”, and each subscale contains four items. The subscales of Intrinsic Motivation To Know (e.g., “Because my studies allow me to learn many interesting things”); Intrinsic Motivation To Succeed (e.g., “For the satisfaction that I feel when I have succeeded in learning difficult academic content); and Intrinsic Motivation To Experience Stimulation (e.g., “Because I really enjoy attending classes”). Responses are provided along a 7-point response format ranging from “1” (“*absolutely doesn’t correspond*”) to “7” (“*corresponds totally*”). Cronbach alpha internal consistency estimates obtained in the present study were .85 for Intrinsic Motivation to Know; .81 for Intrinsic Motivation to Succeed; and .73 for Intrinsic Motivation to Experience Stimulation. Indices of fit obtained through confirmatory factor analysis were χ2/g.l. = 5.8; CFI = .96; IFI = .96; and RMSEA = .068.

#### Approaches to learning

The Revised Questionnaire of Approaches to Learning (RQAL), in its original Spanish language version (Cuestionario Revisado de Procesos de Estudio: *R-CPE-2F*, [37]), was utilized in this study. The instrument contains ten items that assess deep interest in learning with two subscales that assess deep motivation to learn, and deep learning strategies. There is a common stem question across both subscales for each item, “In this class…” for both the deep motivation (DM) subscale (e.g., “Sometimes studying gives me a feeling of deep personal satisfaction”), and deep strategies (DS) subscale (e.g., “I dedicate a lot of my free time reviewing information about interesting themes and concepts that have been covered”). The instrument uses a five-item Likert-type response format ranging from “*Never or almost never true for me*” to “*Always*, *or the majority of the time*, *it is true for me*”. Obtained fit indices were: *χ2/g*.*l*. = 4.6; *CFI* = .94; *IFI* = .94; *RMSEA* = .59.

#### Perceived professional competence

The Perception of Professional Competence Scale developed by Moreno-Murcia, and Silveira [31] was used in the present study. The purpose of the instrument is to assess students’ perceptions of the relevance of their academic knowledge to their anticipated future career, and workplace demands. Responses were completed in relation to the common stem question of, “What my instructors are teaching will permit me to be capable of …”, and a sample item is, “to understand the structure, function, and unique phases of my academic learning”. Responses are provided along a 7-point format ranging from “*completely disagree*” to “*completely agree*”. A Cronbach alpha value of .89 was obtained for the scale in the present study. Indices of fit obtained for this instrument were: χ2/g.l. = 1.7; *CFI* = .99; *IFI* = .99; *RMSEA* = .026.

#### Life satisfaction

The Life Satisfaction Scale (L’Échelle de Satisfaction de Vie) de Vallerand et al. [35], and validated in the Spanish language, and cultural context by Atienza, and colleagues [38] was employed for this study. Participants responded to items that contain a common stem phrase of “Satisfaction with your life…” in relation to five items that represent a single factor (e.g., “In general, my life corresponds with my ideals”. A seven-point response format is used that ranges from “*totally disagree*” to “*totally agree*”. Internal consistency estimate for this instrument was .83, and the obtained indices of fit were: χ2/g.l. = 1.6; *CFI* = .99; *IFI* = .99; *RMSEA* = .025.

### Procedure

Contact was made first with the instructors to inform them of the objectives of the study, and to request their permission to allow their students to complete the questionnaires during class time during required courses. The purpose of the study was explained in a generic way to the participants, and researchers were present to help address any issues that may have been present during the process. Participants were informed that their involvement was entirely voluntary, and that they could discontinue their involvement at any time. Students typically required about twenty minutes to complete the questionnaire. The studies involving human participants were reviewed, and approved by the Ethics commitee of Miguel Hernández University. The participants provided their written informed consent to participate in this study.

### Data analysis

The 1,048 students in the sample were distributed in a similar way in the first 4 years of psychology, and sports programs, from four different universities (250 for the first semester, 280 for the second, 265 for the third, and 253 for the fourth semester). Descriptive analyzes were performed. In order to provide a test of the proposed model, structural equation modeling was used to assess the fit of a model that tested relationships among student autonomy support, psychological need satisfaction, intrinsic motivation in academics, workplace competence, and life satisfaction. To verify the relationship between these variables, the two-step method was used. To perform the analysis of the measurement model, and test the structural equation model, the number of latent variables of the factors was reduced. A confirmatory factor analysis (CFA) was performed on the measurement model, which confirmed the factorial structure of the scales, and tested their construct validity. In the second step, an analysis of structural structures was carried out to measure the predictive power of autonomy support in relation to the other variables. The statistical packages of SPSS 25.0 and AMOS 24 were used.

## Results

Means and standard deviations were computed for all variables, and are provided in Table 1. The mean for instructor autonomy support was 2.39 which is near the midpoint of the scale’s range. Correlations among variables were also computed, and significant relationships existed among each set of variables. Regarding internal consistency, Cronbach’s alpha values for all the variables was between .68, and .89.

**Table 1:** Mean, standard deviation, and correlations between variables.

	*M*	*SD*	α	R	1	2	3	4	5	6	7	8	9	10
**1. Autonomy support**	2.39	.68	.81	4	-	.21**	.33**	.13**	.18**	.21**	.16**	.37**	.21**	.11**
**2. Competence**	3.97	.58	.76	5	-	-	.39**	.49**	.34**	.37**	.33**	.36**	.32**	.34**
**3. Autonomy**	3.17	.72	.68	5	-	-	-	.30**	.15**	.19**	.23**	.34**	.17**	.25**
**4. Relatedness**	4.24	.60	.80	5	-	-	-	-	.20**	.22**	.24**	.26**	.08**	.23**
**5. IM knowledge**	5.06	1.09	.85	7	-	-	-	-	-	.68**	.56**	.42**	.52**	.27**
**6. IM achievement**	4.98	1.13	.81	7	-	-	-	-	-	-	.53**	.50**	.45**	.30**
**7. IM experiences**	4.26	1.24	.73	7	-	-	-	-	-	-	-	.39**	.40**	.26**
**8. Job competence**	4.89	1.02	.89	7	-	-	-	-	-	-	-	-	.33**	.36**
**9. Deep motivation**	2.96	.59	.77	5	-	-	-	-	-	-	-	-	-	.18**
**10. Satisfaction with life**	5.37	1.00	.83	7	-	-	-	-	-	-	-	-	-	-

Note

** *p* < .001; IM: intrinsic motivation.

### Structural equation model of measurement

The proposed model was assessed to determine if the number of latent variables could be reduced on some of the factors. Specifically, to analyze the relationships, and interactions between the variables of the model that is proposed (autonomy support, basic psychological needs (autonomy, competence, relatedness, intrinsic motivation knowledge, intrinsic motivation achievement, intrinsic motivation experiences, job competence, deep motivation, and satisfaction with life), the structural equation model was used.

A series of indices were taken into account [*χ*2, *χ*2/d.f. = l, CFI (comparative fit index), NFI (normed fit index), TLI (Tucker Lewis index), and RMSEA (root mean square error of approximation)].

All the variables showed suitable skewness, and kurtosis values. Also Mardia’s multivariate index was found above 70, so it can be inferred that there was no multivariate normality [39]. The maximum likelihood estimation method, and the covariance matrix between the items were used as input for data analysis. The indices obtained after the analysis were suitable for *χ*2; *p*; *χ*2/d.f.; NFI; CFI; TLI; and RMSEA. These data adjust to the established parameters, so the proposed model can be accepted as good [40]. In the same way, the contribution of the factors to the prediction of other variables was examined using standardized regression weights, which were also suitable.

The result was that the autonomy support factor remained comprised of four items, and basic psychological needs remained comprised of three factors (competence, autonomy, and relatedness) with five items contributing to each. The intrinsic motivation construct that represented self-determined motivation consisted of the three factors (intrinsic motivation to succeed, intrinsic motivation to know, and intrinsic motivation to experience stimulation), and four items represented each factor. The deep learning construct remained unchanged, and consisted of the two factors, motivation, and strategies, with each variable consisting of five measured items. Finally, the social competence, and life satisfaction factors were comprised of eight items, and five items, respectively as in their original structure.

### Test structural regression model

**Fig 1:**
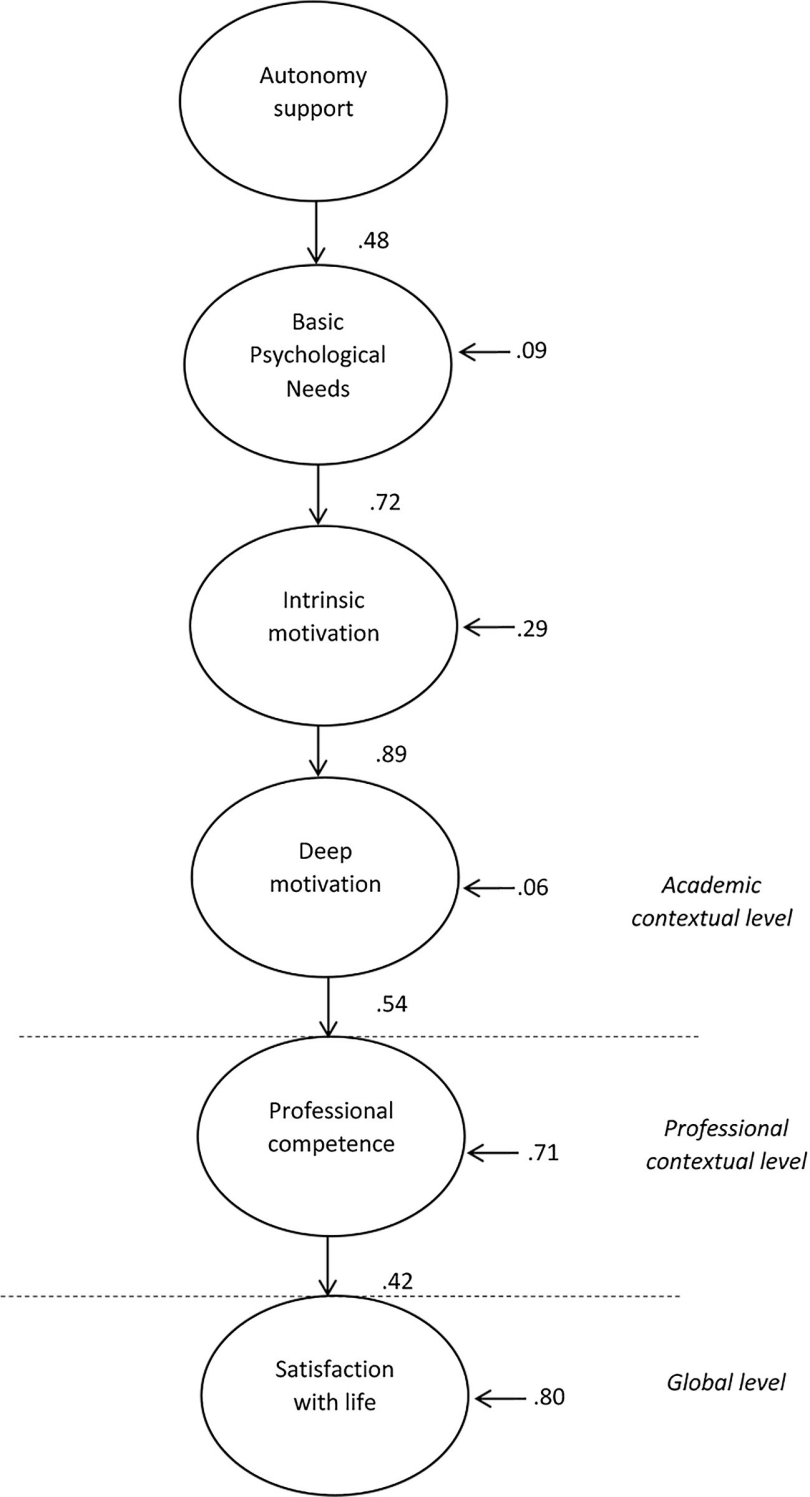
Structural equation model. Parameters are significant at *p* < .05 and standardized.

The maximum versimilitude procedure along with bootstrapping methods were employed. The indices obtained after the analysis presented an adequate adjustment model (χ2 = 9469.5, *p* < 0.01, *χ2/d*.*f*. = 3.81, *CFI* = .94, *IFI* = .94, *TLI* = .92, *RMSEA* = .05), and revealed a positive relationship between instructor autonomy support, and student basic psychological need satisfaction which, in turn, was related to intrinsic motivation, and, consequently, a focus on deeper learning. The deeper learning variable predicted perceived career competence which, in turn, contributed to the explanation of life satisfaction (Fig 1).

### Analysis of measurement invariance by sex

In the analysis of invariance across sex, the objective was to establish whether the structure of the confirmatory factor analysis was invariant in two independent subsamples, one of men, and the other of women, by means of a multigroup analysis. The results showed that the models compared had good fit indices. After the analysis, the differences found between the models were not significant, wich allows establishing a minimum acceptable criterion to consider the existence of invariance in the measurement model.

## Discussion

To date, a limited body of knowledge has been acquired from a self-determination theory perspective in relation to the influence of instructor interpersonal style on student learning approaches, and life satisfaction. The purpose of this study was to examine the predictive capacity of a model that examined the influence of instructor characteristics on student learning approaches, and life satisfaction through a model in which basic psychological need satisfaction, and intrinsic motivation were proposed as mediators. The results provided support for the proposed pattern of expectations.

With regard to the relationship between autonomy support, and basic psychological need satisfaction, and, subsequently, intrinsic motivation, this investigation revealed that autonomy support served as a nutriment for basic psychological need satisfaction, and resulted in adaptive consequences in terms of greater participation, confidence, and commitment by these students, and contributed to a positive relationship with intrinsic motivation. This pattern of results has commonalities with previous research in this area [10, 13, 15, 16, 20]. The implication of these findings is that students benefit when instructors design learning opportunities for which student have opportunities for choice, and opportunities for positive interpersonal relationships. This instructional approach has been linked to a more self-regulated form of learning that can contribute to greater student success [41]. The results of this study also revealed a positive relationship between intrinsic motivation, and a deeper approach to learning, and is consistent with research that indicates that autonomous learning can be enhanced in this way as opposed to a learning strategy that is primarily reliant upon memorization, and repetition [16, 25]. This approach to learning is also linked to the development of competencies, and capacities that allow for stable learning approaches that are dedicated to a more active, and deeper learning approach as well as to a more favorable perception of one’s future professional abilities. Although research in this regard is limited, the findings strengthen the expectation that instructor autonomy support has extensive benefits for student learning processes [27].

The relationship that was proposed in the structural equation model between perceived professional competence, and life satisfaction revealed the presence of a significant, positive relationship between these two variables. No known previous research has been conducted on this relationship, but this outcome is consistent with the focus of self-determination theory in that psychological well-being is anticipated to result when individuals experience feelings of autonomy, and competence [42].

Previous research has indicated that instructor support of student autonomy is related to greater perceived social, and professional competence [43, 44], and can manifest in a more general sense of life satisfaction [45] that may lead to greater student self-confidence about their future occupational roles in society. Some studies in this line of research have examined whether subjective well-being, as an indicator of life satisfaction [46], is positively associated with basic psychological need satisfaction, autonomous motivation, and perceptions of competence [47]. The results that we have obtained reinforce the expectation that motivational benefits that accrue from autonomy support also augment life satisfaction [48, 49]. As such, educators should search for classroom strategies that inspire participation, and creativity during the assimilation of knowledge, and not only satisfy basic psychological needs but also mobilize the student to seek knowledge in a more active manner that can have the effect of contributing to an enduring learning approach that is dedicated to deep learning [30]. In such circumstances, the student may feel that they have the capacities to deal with any of the academic, and professional demands that they confront [21, 31], and may derive greater life satisfaction in the process. In this regard, it is important to highlight the transcontextual interactions that exist within self-determination theory, and Vallerand’s motivation model [50]. In this case, there was evidence of a transcontextual effect from the academic environment (focus on deep learning) with professional consequences (perception of professional competence), and an additional relationship with life satisfaction.

It should be acknowledged that there are limitations to this study. First of all, this is was a cross-sectional study, and so causal relationships cannot be presumed to exist among the variables assessed. Additional experimental, and longitudinal studies would be beneficial to provide a test of the strength of the relationships among instructor autonomy support, and student learning, and life satisfaction outcomes to provide a stronger test of these suppositions. In addition, the structural equation model that was proposed was only one of the possible frameworks for understanding the pattern of relationships among the variables.

As a conclusion, we can point out that the results of this work have clear pedagogical implications as they highlight the benefits that students accrue when they have instructors who encourage them to take a proactive role in the learning process. In this way, instructors can stimulate students’ willingness to initiate the learning process, and can contribute to students’ desire to gain deeper knowledge, and to have the satisfaction of feeling that the knowledge that they acquire will serve them well in the work force, and contribute to their life satisfaction. In this way, both for academic success, and future job potential, as well as for personal well-being, from their classroom practices, teachers have the challenge of being promoters of the intrinsic motivation of their students through an interpersonal teaching style of autonomy support. Consequently, this means that they must be trained in this type of strategy, and be open to pedagogical, and didactic paradigms that go beyond the classic control models to achieve the expected objectives. For HEIs, there is also the challenge of rethinking the direction of the commitment to teacher training, complementing the formal one at the postgraduate level with the pedagogical, and personal one in the direction proposed by the perspective of support for autonomy. In this way, they will not only train successful professionals, but also happy people for a world that today is committed to sustainability, and well-being.
